# Bacteria weigh up costs and benefits of mobile weapons

**DOI:** 10.7554/eLife.111000

**Published:** 2026-03-23

**Authors:** Zhao Wang, Yang Fu

**Affiliations:** 1 Department of Biochemistry, SUSTech Homeostatic Medicine Institute, School of Medicine, Southern University of Science and Technology Shenzhen China

**Keywords:** B. acidifaciens, *P. vulgatus*, gut microbiome, type VI secretion system, T6SS, mobile genetic elements, strain maintenance, Other

## Abstract

Gaining the ability to kill rival microbes is not always an advantage for bacteria in complex gut microbiomes.

**Related research article** Shen BA, Asfahl KL, Lim B, Bertolli SK, Minot SS, Radey MC, Penewit K, Ngo B, Salipante SJ, Johnston CD, Peterson SB, Goodman AL, Mougous JD. 2026. The type VI secretion system governs strain maintenance in a wild mammalian gut microbiome. *eLife* **15**:RP110200. doi: 10.7554/eLife.110200.

Mammals have communities of microbes in their gut that digest food, shape the immune system and protect against pathogens. However, because resources are limited, competition between different species of microbes can be intense. This is why many bacteria have evolved molecular weapons, such as the type VI secretion system (T6SS) – a protein complex that can inject toxins directly into rival cells (Mougous et al., 2006; Pukatzki et al., 2006).

T6SS is widespread among gut bacteria, yet its role remains unclear (Coyne et al., 2016; Verster et al., 2017). Most studies rely on simplified model systems composed of species that do not naturally coexist, leaving doubts about the role of the T6SS in a complex, co-evolved gut microbiome.

Genetic sequences called mobile genetic elements can move within a genome, or even between species. By transferring adaptive traits – such as antibiotic resistance, metabolic functions or systems like T6SS (Figure 1, left) – they accelerate bacterial evolution (Frost et al., 2005). In gut bacteria, clusters of T6SS genes sometimes reside on mobile genetic elements called ICEs (short for integrative and conjugative elements), which facilitate horizontal gene transfer (Johnson and Grossman, 2015). This mobility allows the entire injection apparatus to spread between strains.

**Figure 1.**
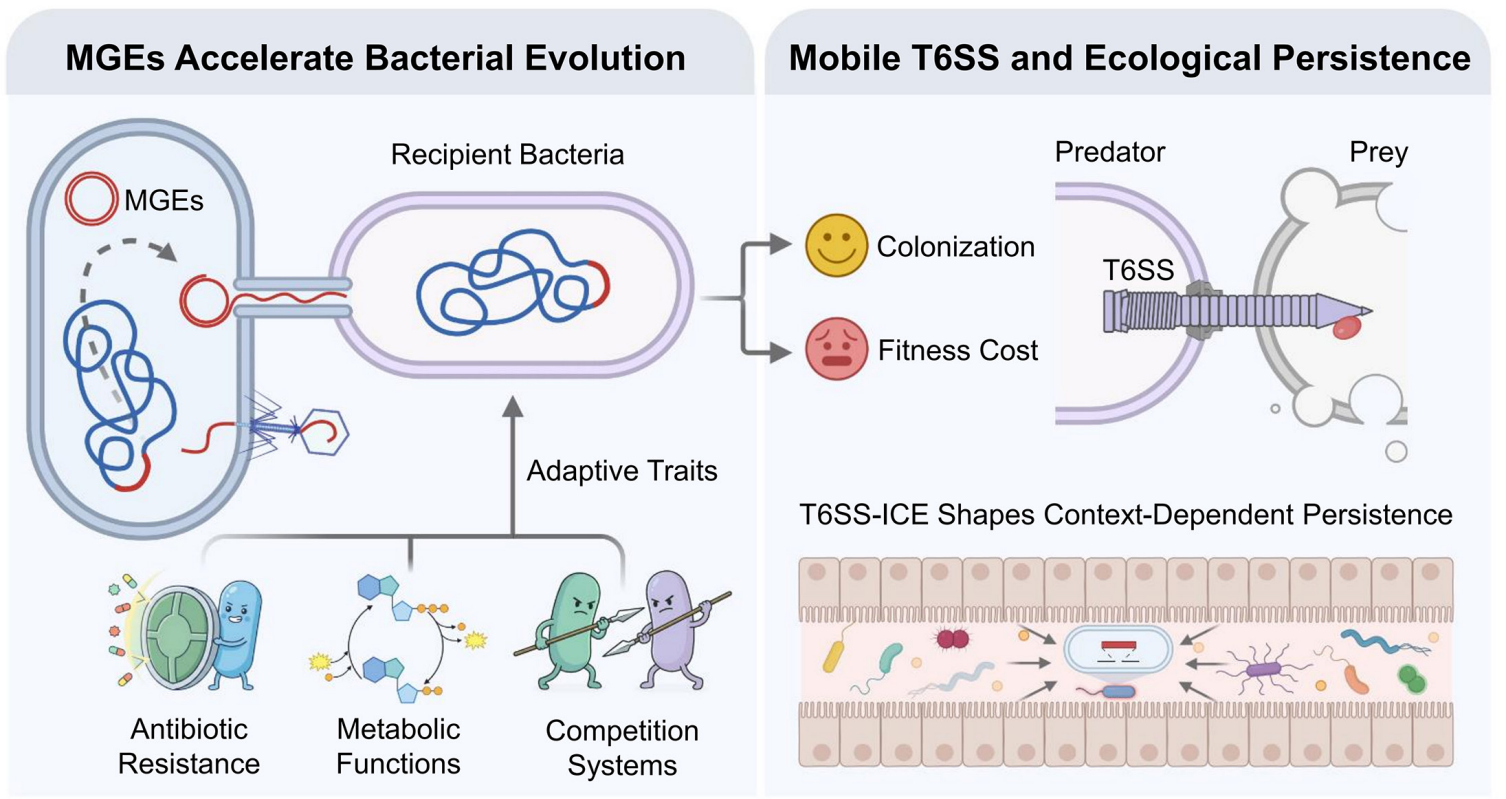
Mobile genetic elements and the gut microbiome. (Left) Mobile genetic elements (MGEs, red circle) facilitate horizontal gene transfer, spreading adaptive traits (such as antibiotic resistance, metabolic functions, and competitive systems like T6SS) among bacteria. (Right) A bacterium with T6SS can attack nearby cells by injecting them with toxins, but maintaining T6SS may incur fitness costs. In complex gut communities, the overall benefit of having TS66 depends on the bacterial species and its physiology and ecology.

However, acquiring mobile genetic elements often comes with a cost. Recent research suggests that mobile genetic elements carrying the genes for T6SS may disrupt the metabolism of the host, or interfere with other mobile elements (Liu et al., 2025). Thus, gaining the genes for T6SS is not always advantageous; any benefits in terms of attacking rival cells must be weighed up against the adverse impact on the metabolism of the host cell itself (Butler and O’Dwyer, 2018). Now, in eLife, Joseph Mougous and colleagues at the University of Washington, Yale University and other institutions in the United States – including Beth Shen as first author – report on how this trade-off plays out in the gut (Shen et al., 2026). The researchers used WildR, a laboratory-propagated microbial community derived from wild mice that has retained much of the diversity and complexity of a natural microbiome (Rosshart et al., 2017).

Shen et al. first sequenced the WildR community. They found that one species, *Bacteroides acidifaciens*, harbors a T6SS on an ICE known to be involved in horizontal gene transfer among closely related bacterial strains. Using genetic tools, they manipulated *B. acidifaciens* within the WildR community to test how the loss of T6SS would affect bacterial fitness. Mutants lacking a functional T6SS colonised the gut efficiently at first, but then declined progressively over time, suffering a reduction of almost two orders of magnitude after prolonged colonization. These findings suggest that T6SS is not essential for the initial establishment of a colony in the gut, but it is critical for long-term survival.

However, not every species benefited equally from T6SS. When the researchers transferred mobile genetic elements carrying T6SS into *Phocaeicola vulgatus*, a bacterium abundant in the WildR community, the system became functional and enabled *P. vulgatus* to kill neighboring bacteria. In mice, however, the transfer imposed a cost: the engineered *P. vulgatus* strain did well to begin with, but was ultimately outcompeted by the native strain, which lacked T6SS (Figure 1 right).

Thus, the cost of sustaining T6SS can outweigh its initial benefits. The balance – shaped by metabolic demands and conflicts with other mobile elements – appears to determine whether this molecular weapon helps or harms its carrier. It remains unclear why the same mobile genetic element can benefit *B. acidifaciens* but harm *P. vulgatus*. One possibility is that *P. vulgatus* lacks the mechanisms needed to regulate T6SS, leading to unnecessary energy expenditure. Alternatively, *B. acidifaciens* and *P. vulgatus* may occupy distinct ecological niches, with only one of these rewarding the ability to kill rival cells. Thus, horizontal gene transfer distributes potential advantage, but actual advantage only emerges if the phenomenon being distributed is compatible with physiology and ecology.

Beyond biological insight, the study of Shen et al. also introduces a powerful method of exploiting the gut’s capacity to replace an existing bacterial strain with a modified version without disrupting community structure. This strategy opens new perspectives for functional studies of complex microbiomes. Moreover, this work reframes bacterial weapons as conditional investments. Mobile genetic elements spread traits, but success hinges on benefits outweighing costs. In complex gut ecosystems, survival depends not just on possessing weapons, but on deploying them at the right cost in the right setting.

## References

[bib1] Butler S, O’Dwyer JP (2018). Stability criteria for complex microbial communities. Nature Communications.

[bib2] Coyne MJ, Roelofs KG, Comstock LE (2016). Type VI secretion systems of human gut Bacteroidales segregate into three genetic architectures, two of which are contained on mobile genetic elements. BMC Genomics.

[bib3] Frost LS, Leplae R, Summers AO, Toussaint A (2005). Mobile genetic elements: the agents of open source evolution. Nature Reviews Microbiology.

[bib4] Johnson CM, Grossman AD (2015). Integrative and conjugative elements (ICEs): what they do and how they work. Annual Review of Genetics.

[bib5] Liu M, Wang H, Wang Z, Wang H, Zhang K, Xue J, Liu R, Liu Y, Xia P, Wang H, Kan B, Li Y, Li S, Fu Y (2025). A *Vibrio*-specific T6SS effector reshapes microbial competition by disrupting *Vibrio* bioenergetics. Cell Host & Microbe.

[bib6] Mougous JD, Cuff ME, Raunser S, Shen A, Zhou M, Gifford CA, Goodman AL, Joachimiak G, Ordoñez CL, Lory S, Walz T, Joachimiak A, Mekalanos JJ (2006). A virulence locus of *Pseudomonas aeruginosa* encodes a protein secretion apparatus. Science.

[bib7] Pukatzki S, Ma AT, Sturtevant D, Krastins B, Sarracino D, Nelson WC, Heidelberg JF, Mekalanos JJ (2006). Identification of a conserved bacterial protein secretion system in *Vibrio cholerae* using the *Dictyostelium* host model system. PNAS.

[bib8] Rosshart SP, Vassallo BG, Angeletti D, Hutchinson DS, Morgan AP, Takeda K, Hickman HD, McCulloch JA, Badger JH, Ajami NJ, Trinchieri G, Pardo-Manuel de Villena F, Yewdell JW, Rehermann B (2017). Wild mouse gut microbiota promotes host fitness and improves disease resistance. Cell.

[bib9] Shen BA, Asfahl KL, Lim B, Bertolli SK, Minot SS, Radey MC, Penewit K, Ngo B, Salipante SJ, Johnston CD, Peterson SB, Goodman AL, Mougous JD (2026). The type VI secretion system governs strain maintenance in a wild mammalian gut microbiome. eLife.

[bib10] Verster AJ, Ross BD, Radey MC, Bao Y, Goodman AL, Mougous JD, Borenstein E (2017). The landscape of type VI secretion across human gut microbiomes reveals its role in community composition. Cell Host & Microbe.

